# A Time–Frequency-Based Data-Driven Approach for Structural Damage Identification and Its Application to a Cable-Stayed Bridge Specimen

**DOI:** 10.3390/s24248007

**Published:** 2024-12-15

**Authors:** Naiwei Lu, Yiru Liu, Jian Cui, Xiangyuan Xiao, Yuan Luo, Mohammad Noori

**Affiliations:** 1 School of Civil Engineering, Changsha University of Science and Technology, Changsha 410114, China; lunaiwei@csust.edu.cn (N.L.); cuijian@stu.csust.edu.cn (J.C.); xiangyuan@stu.csust.edu.cn (X.X.); 2 College of Civil Engineering, Hunan University of Technology, Zhuzhou 412007, China; luoyuan@hut.edu.cn; 3 Department of Mechanical Engineering, California Polytechnic State University, San Luis Obispo, CA 93407, USA; mnoori52@yahoo.com; 4 School of Civil Engineering, University of Leeds, Leeds LS2 9JT, UK

**Keywords:** structural damage identification, structural health monitoring, convolutional neural network, gram angle difference field, cable-stayed bridge

## Abstract

Structural damage identification based on structural health monitoring (SHM) data and machine learning (ML) is currently a rapidly developing research area in structural engineering. Traditional machine learning techniques rely heavily on feature extraction, where weak feature extraction can lead to suboptimal features and poor classification performance. In contrast, ML-based methods, particularly deep learning approaches like convolutional neural networks (CNNs), automatically extract relevant features from raw data, improving the accuracy and adaptability of the damage identification process. This study developed a time–frequency-based data-driven approach aiming to improve the effectiveness of traditional data-driven structural damage identification approaches for large complex structures. Firstly, the structural acceleration signals in the time domain were converted into two-dimensional images via the Gram angle difference field (GADF). Subsequently, the characteristic feature in the image data was studied by convolutional neural networks (CNNs) to predict the structural damage conditions. An experimental study on a scale model of a cable-stayed bridge was conducted to identify the damage of stay cables under the moving vehicle load on the main girders. The CNN was employed to extract the characteristic features from the time-varying monitoring data of vehicle–bridge interactions. The CNN parameters were optimized to conduct the structural damage classification task. The performance of the proposed method was evaluated by comparing it with various traditional pre-trained networks. The effect of environmental noise on the prediction accuracy was also investigated. The numerical results show that the ResNet model has the best performance in terms of damage identification accuracy and convergence speed, achieving higher accuracy and faster convergence compared to the other four traditional networks. The method can accurately identify damage on bridges using insufficient sensors on the bridge deck, which has valuable potential for application to real-world bridges with monitoring data. As the Signal-to-Noise Ratio (SNR) decreases from 20 dB to 2.5 dB, the prediction accuracy of ResNet decreases from 86.63% to 62.5%, which demonstrates the robustness and reliability in identifying structural damage.

## 1. Introduction

In recent decades, numerous long-span bridges have been constructed with the rapid development of highway infrastructure and the global economy. In addition, more challenging cable-supported bridges spanning a sea or canyon are being built or planned. In long-term complex environments, structural components such as cables and girders are prone to damage, leading to structural performance degradation and durability problems [1,2,3]. Cable-supported bridges typically have complex structural systems characterized by strong nonlinearity, high-order redundancy, time variability, and susceptibility to damage. Traditional physical-driven methods, such as modal analysis, have limitations in identifying damage in complex bridge structures [4,5,6,7,8]. With the advancement of structural health monitoring (SHM) technology and computer technology, artificial intelligence and data-driven methods show great advantages for structural damage identification and safety assessment for complex structures [9,10]. Currently, the infrastructure operation and maintenance industry is entering the big data era. Therefore, it is crucial to fully extract the SHM data in order to perform damage identification for complex infrastructures.

Structural dynamic responses contain modal parameters such as natural frequencies and mode shapes, which are sensitive to structural elements, boundary conditions, and joints [11]. Given that minor structural damage can significantly alter dynamic responses, damage identification based on modal analysis of vibration data from the SHM system should be widely applicable [12]. However, the measurable modal parameters are often insensitive to such damage because structural damage is typically a localized phenomenon. The primary live load on bridge structures during their service life is moving vehicle loads, where a vehicle passing over a damaged section of the bridge produces a localized dynamic response [13,14,15]. Damage detection methods based on the interaction response between the bridge and moving vehicles are highly sensitive to detecting local damage [16]. Considering that traffic loading is often unknown in practice, Zhu [17] proposed a method for simultaneously identifying structural damage and moving vehicle loads. Li et al. [18] also introduced a bridge structure damage detection method based on the reconstruction of the bridge dynamic response without requiring any prior knowledge of the moving vehicles.

Structural damage identification based on SHM data and machine learning is currently a research hotspot in the field of structural engineering. Traditional machine learning techniques usually rely on feature extraction, where weak feature extractions can lead to insufficient deficiency and poor classification performance [19]. To overcome this problem, deep learning methods are powerful tools that enable the autonomous extraction of optimal features from raw data. Therefore, the deep learning method has higher classification and regression performance compared with traditional machine learning [20]. Embedding feature extraction into deep learning networks can improve the efficiency of structural damage detection and identification [21]. Machine learning-based methods offer several notable advantages over traditional model-based techniques, particularly in damage identification and monitoring. ML methods can autonomously extract relevant features from raw sensor data, eliminating the need for manual feature selection and prior knowledge of the system’s behavior. In contrast to classical methods, which often struggle with nonlinear relationships, ML algorithms excel at modeling complex, nonlinear damage patterns, thereby enhancing accuracy across a wide range of scenarios. Furthermore, with real-time data processing capabilities, ML approaches facilitate continuous, adaptive damage detection, which is essential for proactive maintenance and ensuring structural safety. There are three main deep learning methods for structural damage identification: deep autoencoders, convolutional neural networks (CNNs), and recurrent neural networks (RNNs) [22,23]. CNNs consist of multiple convolutional layers, each followed by a pooling layer. The convolutional and pooling layers are used for feature extraction and dimensionality reduction, automatically extracting high-level features from raw signals or low-level features, followed by fully connected layers for classification purposes [24]. However, training deep learning networks requires a large amount of high-quality labeled data [25], and obtaining labeled damage data from real bridges is challenging. Thus, the application of deep learning approaches in bridge damage detection is still challenging.

Research progress has been made to address the challenge of damage identification of bridges based on monitoring data. Singh et al. [26] proposed a hybrid method combining empirical mode decompositions based on time-varying filtering with synchronous extraction transformation to track the time-varying modal parameters of bridges. This method uses vehicle–bridge coupled vibration responses to identify structural damage and introduces the vehicle scanning method. Pan et al. [27] identified bridge damage through signal discontinuities in structural dynamic responses. Wang et al. [28] analyzed the noise resistance and robustness of CNNs in feature extraction and improved the recognition performance under different noise intensities. Haji Alizadeh [29] conducted structural damage identification research using CNNs based on a simply supported beam numerical model and validated the identification accuracy of damage location and extent using the numerical model. Fernandez-Navamuel et al. [30] proposed a method to introduce the effect of environmental and operational conditions into the synthetic damage scenarios employed for training a deep neural network, which is applicable to large-scale complex structures. Zhang et al. [31] proposed a methodology that applies pattern recognition methods to guide Bayesian model updating (BMU) and supervise the identification of structural damage. Yang et al. [32] used a recursive analysis method to present structural damage features in the form of recurrence plots and achieved good damage identification results. Silik et al. [33] analyzed SHM data based on wavelet time–frequency features, revealing sensitive features for structural damage extraction. However, the effectiveness of bridge damage identification methods based on insufficient monitoring data is still limited.

This study developed a time–frequency-based data-driven approach aiming to improve the effectiveness of traditional data-driven approaches for structural damage identification in large complex bridges. Firstly, an image encoding technique was utilized to transfer vehicle–bridge vibration acceleration responses into the time–frequency domain, and then a labeled training dataset using the time–frequency images was constructed. Subsequently, the proposed approach was compared with four pre-trained CNN models including the SVM, the GoogLeNet, the ResNet-34, and the AlexNet. Model hyperparameters were optimized for each classifier to achieve optimal performance. In addition, the damage classification performance of each classifier was compared based on their best results. The pre-trained network and optimized classifier combination with the best performance were selected, and the impact of noisy acceleration data on the proposed method performance was analyzed. The findings provide theoretical support for bridge structural damage identification based on health monitoring data, particularly contributing to improved identification accuracy and the enhanced reliability of damage detection.

## 2. Theoretical Basis of Structural Damage Identification Based on CNNs

### 2.1. GADF Theory

Gramian angular field (GAF) is a technique for encoding time series data into two-dimensional images based on coordinate transformations and Gram matrices [34]. GAF converts the time series into polar coordinates and then applies the Gram matrix to transform the resulting angles. Although the Gram matrix preserves temporal dependencies, it can balance between valuable information and Gaussian noise. A spatial transformation is typically applied by converting Cartesian coordinates into polar coordinates [35].

In the Cartesian coordinate system, whether rectangular or oblique, two coordinate axes intersect at the origin to create a plane affine coordinate system, where the metric units of both axes are consistent. Acceleration data are typically represented in a two-dimensional rectangular coordinate system, where the position of each point is defined by two numerical values. The polar coordinate system, another two-dimensional system, defines a point as the pole and a ray from this point as the polar axis, with a specified unit length and angle in the positive direction. In the polar coordinate system, a position is represented by its distance from the origin and the angle relative to the polar axis. As a result, points in the Cartesian coordinate system can be directly mapped to corresponding points in the polar coordinate system.

The Gram matrix, frequently used in linear algebra and geometry, calculates the linear relationships among a set of vectors. For a set of *n* vectors, the Gram matrix consists of the pairwise dot products of the vectors:

(1) $$\left(\begin{array}{cccc} \langle u_{1},v_{1}\rangle & \langle u_{1},v_{2}\rangle & \cdots & \langle u_{1},v_{n}\rangle \\ \langle u_{2},v_{1}\rangle & \langle u_{2},v_{2}\rangle & \cdots & \langle u_{2},v_{n}\rangle \\ \vdots & \vdots & \ddots & \vdots \\ \langle u_{n},v_{1}\rangle & \langle u_{n},v_{2}\rangle & \cdots & \langle u_{n},v_{n}\rangle \end{array}\right)$$

where *u_i_* and represents the distance of the *i*-th component, and *v_i_* represents the angle of the *i*-th component. For the special condition that the norms of vectors *u* and *v* are both equal to one, the dot product of the two vectors can be expressed by

(2) $$\langle u,v\rangle =\cos(\theta )$$

where *θ* represents the angle between vectors *u* and *v*.

If these vectors are unit vectors, their dot product depends entirely on the angle between them. This angle is expressed in radians, leading to a value ranging from minus one to one. Assuming the vectors are unit vectors, the Gram matrix simplifies to

(3) $$G=\left(\begin{array}{cccc} \cos(\theta_{1,1}) & \cos(\theta_{1,2}) & \cdots & \cos(\theta_{1,n})\\ \cos(\theta_{2,1}) & \cos(\theta_{2,2}) & \cdots & \cos(\theta_{2,n})\\ \vdots & \vdots & \ddots & \vdots \\ \cos(\theta_{n,1}) & \cos(\theta_{n,2}) & \cdots & \cos(\theta_{n,n}) \end{array}\right)$$

where $\theta_{i,j}$ represents the angle between the *i*-th and *j*-th unit vectors.

The acceleration response of a cable-stayed bridge is typically represented as a one-dimensional time series. In the Cartesian coordinate system, the horizontal axis represents time, and the vertical axis shows the magnitude of acceleration at each time point, with units of m/s^2^.

Suppose the acceleration data collected by the sensor is *X* = {$x_{1}$, $x_{2}$, $x_{3}$, ……, $x_{n-1}$, $x_{n}$}, where *x_i_* denotes the *i*-th acceleration value. First, the data are normalized and scaled to the range [−1, 1] using the following common formula:

(4) $$x_{i}^{\prime }=\frac{x_{i}-\max X+x_{i}-\min X}{\max X-\min X}$$

where $x_{i}^{\prime }$ represents the data value after normalization.

Second, transform the scaled acceleration time series into polar coordinates. In polar coordinates, acceleration values are represented as angles and timestamps. To encode in polar coordinates, the scaled acceleration values are represented using the cosine of the angle, with angles ranging [0, π]. The timestamps are represented as the radius. The formula is written as

(5) $$\left\{\begin{array}{c} \begin{array}{c} \theta_{i}=\arccos(x_{i}^{\prime })-1\leq x_{i}^{\prime }\leq 1,x_{i}^{\prime }\in X^{\prime }\\ M=\frac{1}{\max X-\min X} \end{array}\\ r_{i}=\frac{t_{i}}{M} \end{array}\right.$$

where *t*_1_ denotes the timestamp, and *M* represents the normalization factor.

The interval [0, 1] is segmented, with zero being excluded. The remaining points are mapped to the time series data, ensuring that each point is positioned within a unit circle in polar coordinates. The acceleration time series will progressively distort within this unit circle, varying in angles and radii. The polar transformation from the previous step includes temporal information, enabling the use of GAF to reconstruct the acceleration time series and produce the GADF, as described by

(6) $$A_{GADF}(i,j)=[\sin(\theta_{i}-\theta_{j})]=\left(\begin{array}{c} \sin(\theta_{1}-\theta_{1})\cdots \sin(\theta_{1}-\theta_{j})\\ \sin(\theta_{2}-\theta_{1})\cdots \sin(\theta_{2}-\theta_{j})\\ \vdots \\ \sin(\theta_{i}-\theta_{1})\cdots \sin(\theta_{i}-\theta_{j}) \end{array}\right)$$

where $\theta_{i}$ and $\theta_{j}$ are the angles of the *i*-th and *j*-th data points in the time series.

### 2.2. Training CNNs

Once the images of the acceleration signals are obtained, additional processing and analysis are needed to extract meaningful information and ensure accurate assessments. This study utilized four traditional neural networks, each specifically designed to process and analyze these image data. Each features a unique structure and function, enabling it to interpret and analyze the acceleration signals from different perspectives and at various levels.

A Support Vector Machine (SVM) is a supervised learning algorithm commonly used for classification tasks. It works by identifying the optimal hyperplane that separates data points into distinct classes. In this study, we applied a [linear/nonlinear] kernel SVM for damage classification, using features extracted from sensor data. To improve the performance of the SVM model, we performed input feature selection using [Principal Component Analysis (PCA)/Recursive Feature Elimination (RFE)/Mutual Information]. This approach helped reduce the dimensionality of the data and focus on the most relevant features for classification. Specifically, we selected the top [X] features based on the [method]. The model was trained on the selected features using the training dataset and validated with a separate validation set. SVM performance was evaluated using [accuracy/F1-score/ROC curve], and the results were compared with those of the CNN model to assess the effectiveness of the two approaches.

The second CNN is the AlexNet, which is one of the earliest pioneering implementations of deep convolutional neural networks. It was the first to incorporate ReLU activation and dropout for regularization. Developed by Alex Krizhevsky, Ilya Sutskever, and Geoffrey Hinton in 2012 [36], AlexNet was the first convolutional neural network to win the ImageNet Large Scale Visual Recognition Challenge (ILSVRC).

The third CNN is the GoogLeNet developed by Google in 2014, which won the ImageNet Large Scale Visual Recognition Challenge (ILSVRC) that same year.

**Figure 1:**
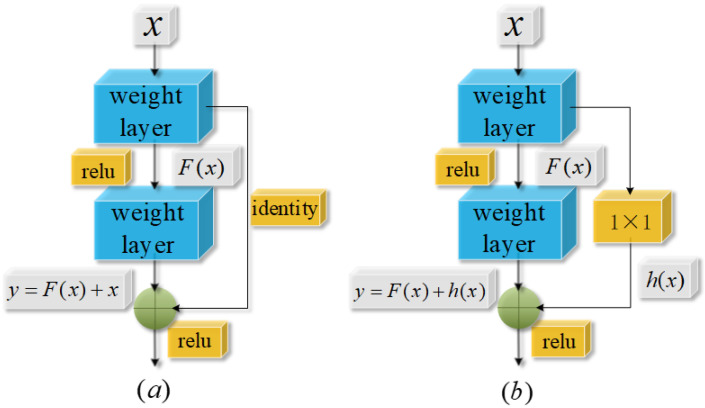
Residual block structure diagram: (**a**) Same; (**b**) Different.

He et al. [37] introduced Residual Networks (ResNets) to overcome the vanishing and exploding gradients encountered in deep neural networks during training. A ResNet consists of several stacked residual blocks, which use skip connections in addition to standard convolutional layers to enhance the learning of residuals. The structure of a residual block is illustrated in Figure 1, where Figure 1a and Figure 1b depict residual blocks with the same and different output feature dimensions compared to the input, respectively.

As shown in Figure 1, the output *y* of a residual block is the sum of the input *x* and the residual mapping output *F*(*x*), where *F*(*x*) is computed by applying convolution and activation functions to *x*. The detailed computation process is written by

(7) $$x^{l}=\sum_{i=1}^{c}w*x+b$$

where *x* is the input to the residual block, *c* represents the number of channels, *ω* is the weight matrix for the convolutional kernels, *b* is the bias term, ∗ denotes the convolution operation, and *x^l^* is the outcome of this convolution [37].

(8) $$y^{l}=f(x^{l})=\max\{0,x^{l}\}$$

where *x^l^* is the feature map resulting from the convolution operation detailed in Equation (8), and *y^l^* is the output after applying the ReLU activation function to *x^l^*.

**Figure 2:**
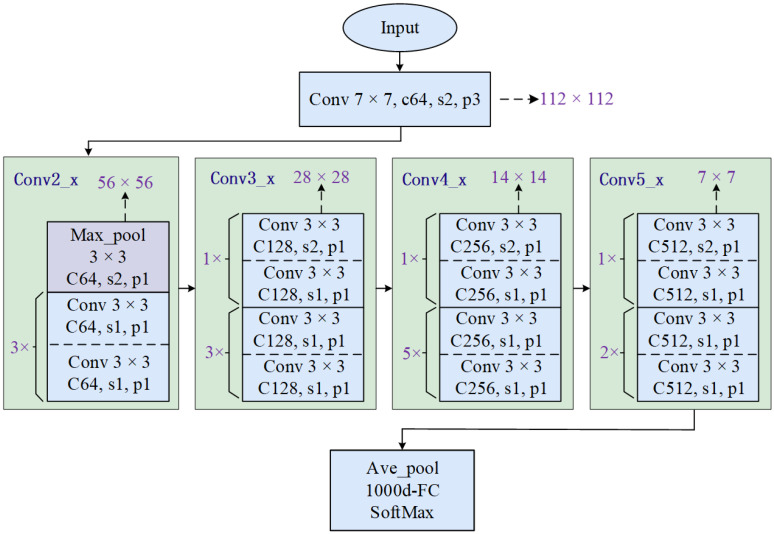
Framework of a typical ResNet-34.

If the number of channels in the input *x* differs from that in the residual mapping output *F*(*x*), a 1 × 1 convolution operation is employed to match their feature dimensions, as illustrated in Figure 1a. The layout of the ResNet is shown in Figure 2.

Once images of the acceleration signals are obtained, the raw acceleration data are converted into image format through preprocessing. The images are then input into four distinct CNNs. Features are extracted from the images through various layers, including the input layer, convolutional layers, activation functions, pooling layers, fully connected layers, and output layers. These features are then used for classification or recognition.

## 3. Model Test Design

### 3.1. Overview of the Cable-Stayed Bridge Specimen

**Figure 3:**
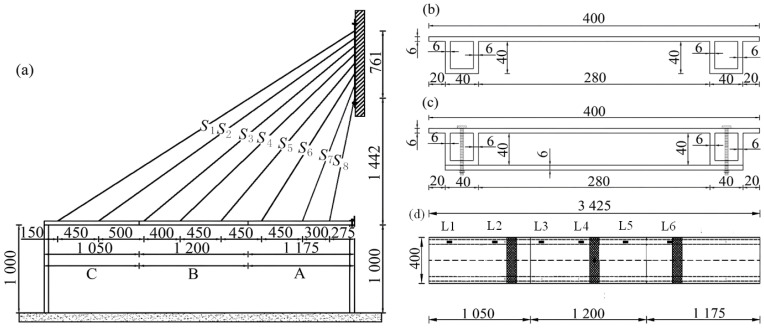
Cable-stayed bridge layout: (**a**) overall layout; (**b**) cross-sectional dimensions; (**c**) spliced section dimensions; (**d**) plan view layout.

A 1/4 scale model was constructed to represent the engineering background of a double-tower concrete cable-stayed bridge. The main girder measures 3.425 m in length (1.175 + 1.200 + 1.050 m) and 0.400 m in width. The main cross-section is a girder with double steel boxes. At the joints of the steel box girder segments, connection steel plates were placed on both the inner and outer sides of the edge box girder. The steel box girder segments were joined using connection steel plates and M12 bolts, creating a continuous steel main girder and maintaining longitudinal integrity. The prototype bridge has 34 pairs of stay cables. Based on similitude, the anchorage spacing on the main girder would be just 0.15 m, making manufacturing impractical. Furthermore, the dense arrangement of stay cables results in minimal variation in cable forces, making it unsuitable for testing the model under typical damage conditions. As a result, the model bridge was simplified to 8 pairs of stay cables. Cables were retained at the edge piers and main towers, with every three adjacent cables combined into one in the middle sections. The arrangement of the model bridge is shown in Figure 3a. The unknown load factor method was used to optimize the cables forces, ensuring that the stresses and deflections at critical sections are at the idealized position. The adjusted design cable forces are shown in Table 1.

**Figure 4:**
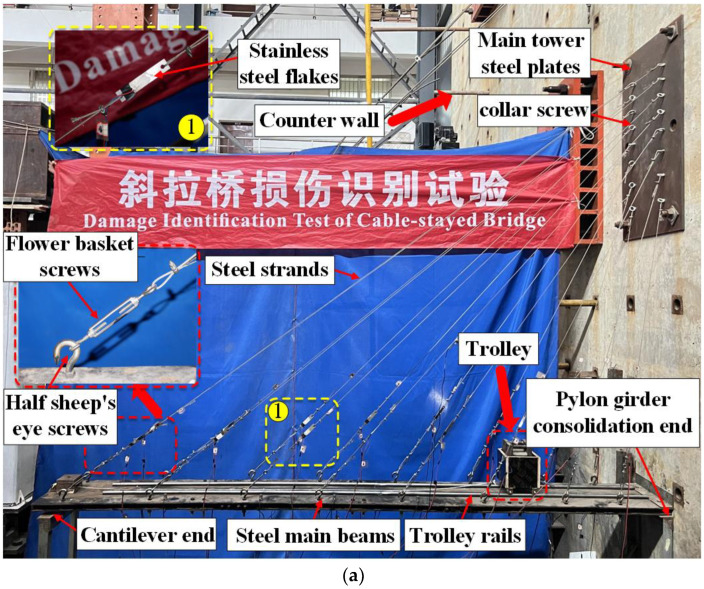

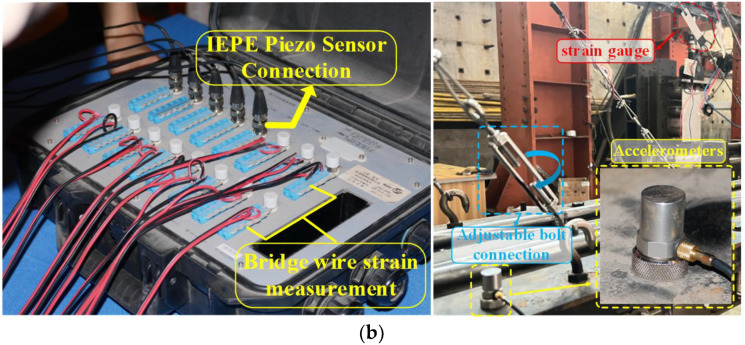
Test details: (**a**) overall; (**b**) details.

The model test loading setup is shown in Figure 4a. The stay cables are fitted with devices for measurement and adjustment of tension. The tension measurement device comprises stainless steel thin plates and strain gauges. Tension in the stay cables was adjusted with basket screws to ensure the desired tightness of the steel cables. The bolts connected the stay cables to the steel main girder. Other sections of the main girder followed the cross-section design shown in Figure 3b. The base was bolted integrally to the steel main girder, as shown in Figure 3c. The main tower of the model bridge was simplified by using steel plates, which were bolted to the reaction wall. The stay cables and main tower steel plates were connected with ring bolts. The cable ends were anchored using cable clamps. The pylons were constructed from angle steel and simulate vertical, horizontal, and longitudinal constraints. Bolted connections between the tower and pier beams were bolted to simulate fixed supports.

### 3.2. Probabilistic Modeling of Traffic Parameters

**Figure 5:**
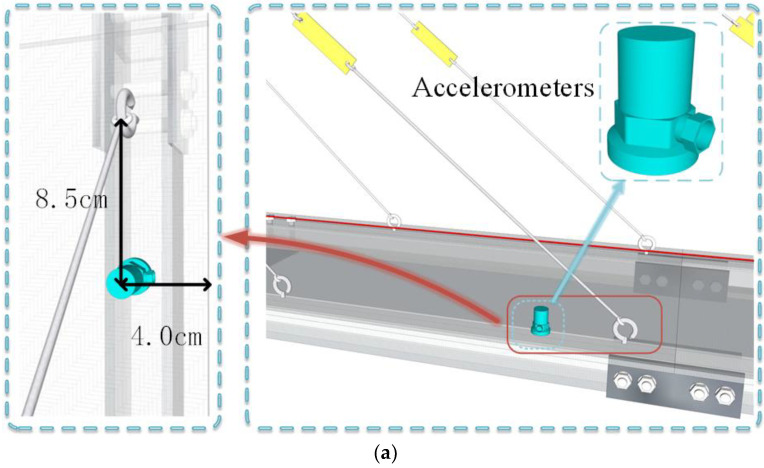

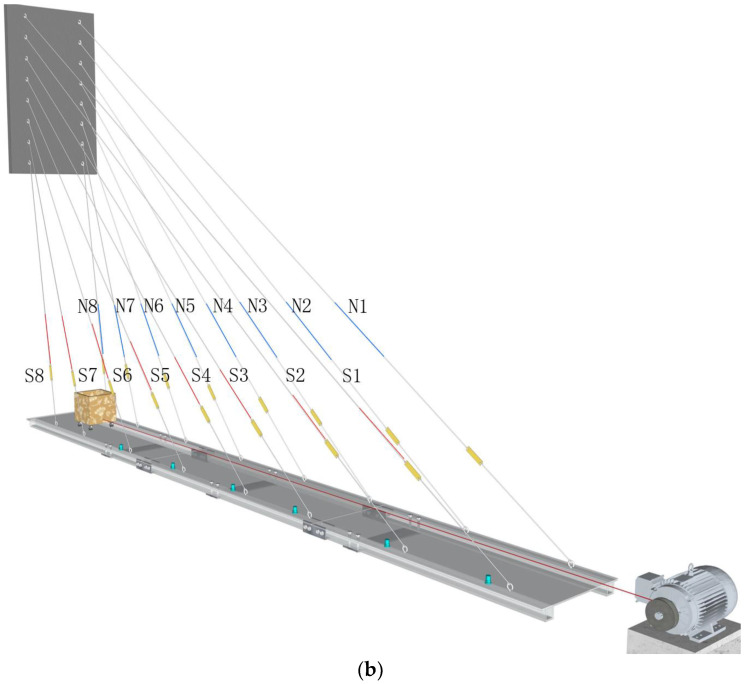
Three-dimensional diagram of damage test arrangement of cable-stayed bridge: (**a**) accelerometer location diagram; (**b**) overall layout diagram.

The cable-stayed bridge model is depicted in Figure 3. Given the model’s small size and mass, challenges such as low response amplitude and high-frequency effects may arise when measuring response signals. Therefore, the added mass method is used to determine the structural flexibility matrix, with a primary focus on identifying the lower-order modes related to structural damage. As a result, fewer acceleration sensors are required. To monitor the variations in the main girder vibrations under different damage conditions, six sets of acceleration sensors, labeled L1 through L6, were installed. The sensor layout is shown in Figure 3d, while Figure 5 shows the details of the specific placement of the acceleration sensors. Response signals are collected by moving a model car at a constant speed, powered by an electric motor. Each data collection session lasts eight seconds with a sampling frequency of 500 kHz. The dynamic response acceleration of the structure is captured using the DH3822 dynamic signal acquisition device. In this experiment, data from all sensors were collected, and the data from all sensors under the same operating condition were placed in a single dataset for that condition.

### 3.3. Damage Condition Setup

To study damage identification in typical scenarios, model testing was performed on a cable-stayed bridge, focusing on cable breakage. The tension in the cables was adjusted using basket screws, and the steel cables were removed to simulate cable damage. Table 2 shows the parameters for each damage condition. A total of eight experimental conditions were designed, including one healthy condition, three single-cable damage conditions, and four multiple-cable damage conditions. Condition 1 represents the healthy state, Conditions 2 to 4 represent single-cable damage, and Conditions 5 to 8 represent multiple-cable damage, with a damage extent of 30% for all cases. This varied experimental setup provides comprehensive coverage of conditions, facilitating a thorough evaluation of the cable-stayed bridge’s response and performance under different damage scenarios. To ensure accurate and consistent results, the cart’s speed, weight, and the placement of acceleration sensors must remain constant across experiments. Maintaining these strict control conditions ensures reliable data, offering a solid scientific basis for damage identification in cable-stayed bridges. We fixed the damage level at 30% to standardize the experimental conditions and ensure a consistent benchmark for comparing the performance of different methods. This choice was made to simplify the analysis and eliminate the additional variability that could arise from using different damage levels. By fixing the damage level, we were able to isolate the effects of the methods themselves, providing a more controlled environment for evaluation. While in real-world scenarios the degree of damage can vary, this approach allowed for a focused comparison under uniform conditions. Future work will explore the adaptability of these methods to varying damage levels.

## 4. Results and Discussions

### 4.1. Time–Frequency Analysis Based on GADF

**Figure 6:**
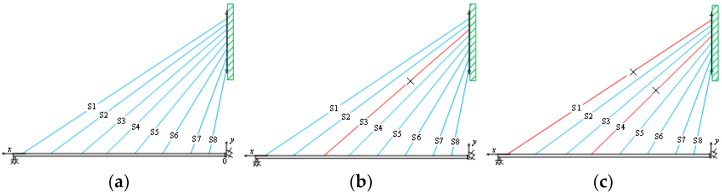
Different damage conditions: (**a**) healthy state; (**b**) single-cable damage; (**c**) multiple-cable damage.

**Figure 7:**
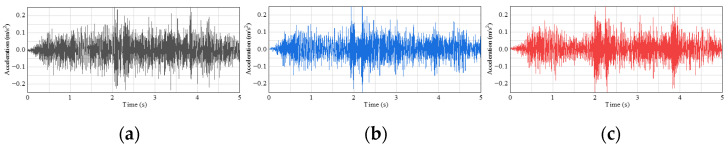
Comparison of time domain signals: (**a**) health condition; (**b**) single-cord damage condition; (**c**) double-cord damage condition.

The vibration acceleration signals generated by the cart moving load under different damage conditions were measured experimentally. To better understand the damage information in the vibration signals and analyze the characteristics under different damage conditions, the experimentally obtained data are presented in the form of time domain plots. Examples of the time domain signals for certain conditions are shown in Figure 6 and Figure 7.

**Figure 8:**
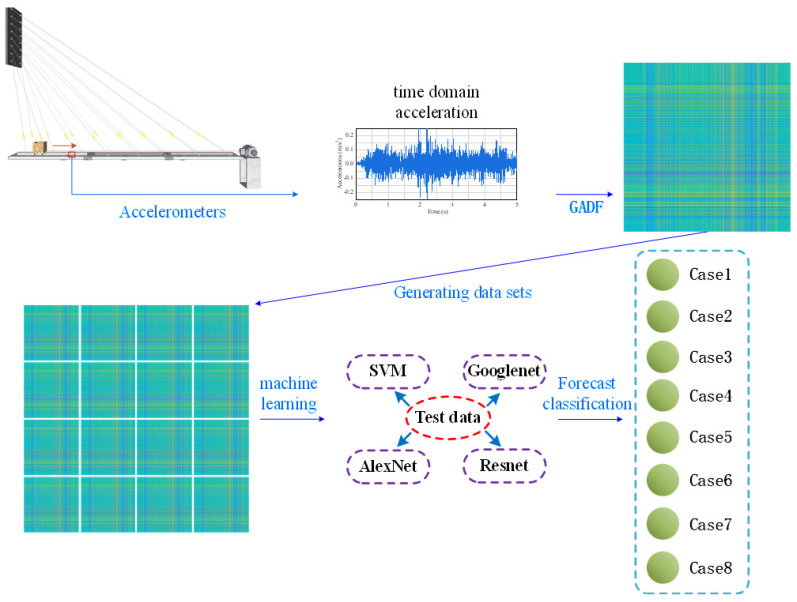
Signal-to-picture classification and recognition flowchart.

In this experiment, to improve classification and analysis accuracy using deep learning methods, the one-dimensional time domain signals were transformed into two-dimensional images using the GADF method. This method retains the temporal dependencies and nonlinear characteristics of the time series within the images. After converting, the images were organized into datasets representing different conditions and analyzed using machine learning for classification. The process of converting signals into images and performing classification is shown in Figure 8.

### 4.2. Comparison with Traditional Networks

**Figure 9:**
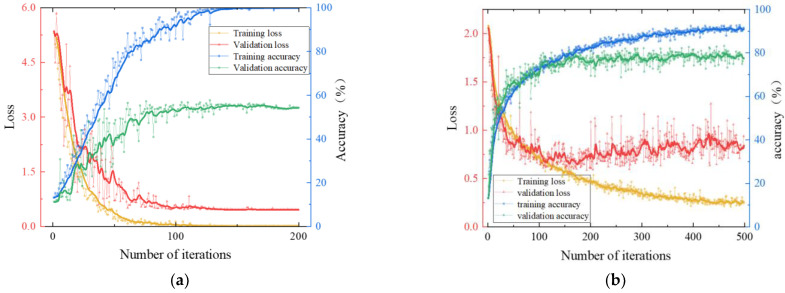

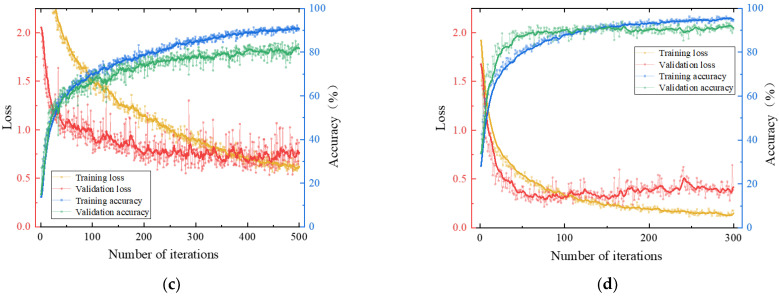
Comparisons of four network recognition under accuracy and damage: (**a**) SVM. (**b**) AlexNet. (**c**) GoogLeNet. (**d**) ResNet.

The vibration signals, transformed into binary images, were split into training, validation, and test sets at a 3:1:1 ratio. The training set consists of 1152 images, while the validation and test sets each contain 384 images. The accuracy of damage identification in four CNN methods was compared. Accuracy refers to the proportion of correctly predicted samples to the total number of samples in the damage identification task. To maintain consistency, all methods used identical hyperparameters, and the accuracy of damage identification was averaged across all cases. After comparing different hyperparameters, we ultimately set the learning rate to 0.0001 and the batch size to 32, which achieved the best damage identification performance. The training processes for the four models are shown in Figure 9.

It can be observed from Figure 9 that the accuracies of different methods are totally different. The SVM stabilizes in validation accuracy and loss after about 130 iterations, showing no significant improvement beyond this point. AlexNet converges relatively slowly, though its accuracy is significantly higher than that of SVM. GoogleNet shows a plateau in validation accuracy around 500 iterations, with an improvement over AlexNet. ResNet converges rapidly, with validation accuracy and loss stabilizing around 80 iterations, achieving higher accuracy than the other three networks and requiring fewer iterations to do so. These observations can be explained as follows: SVM struggles with complex image data, leading to lower accuracy; AlexNet, despite its effective feature extraction, has difficulty capturing deep features due to its relatively shallow architecture; GoogleNet, with its deeper structure and higher accuracy, experiences slower training and potential overfitting due to its complex architecture and high computational demands; ResNet, by using residual blocks, effectively mitigates the vanishing gradient problem in deep networks.

**Figure 10:**
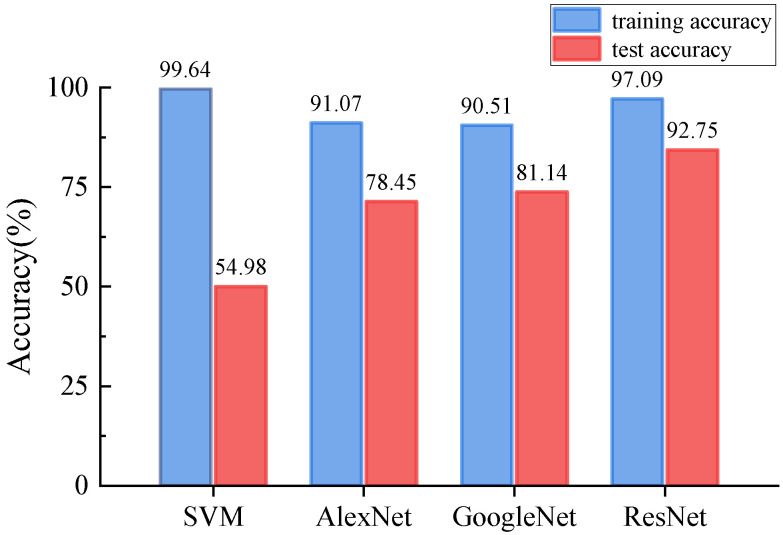
Comparison chart of the accuracy of the four networks.

Figure 10 summarizes the test accuracies of the four methods used in this study. The SVM achieved an average training accuracy of 99.64%, but its test accuracy was much lower, at 54.98%, reflecting relatively poor performance. In contrast, AlexNet showed an average training accuracy of 91.07% and a test accuracy of 78.45%, representing a significant improvement over SVM. GoogleNet attained an average training accuracy of 90.51% and a test accuracy of 81.14%, slightly outperforming AlexNet. ResNet achieved the highest performance, with an average training accuracy of 97.09% and a test accuracy of 92.75%. ResNet’s test accuracy notably exceeds that of SVM, AlexNet, and GoogleNet by 37.77%, 14.3%, and 11.61%, respectively.

**Figure 11:**
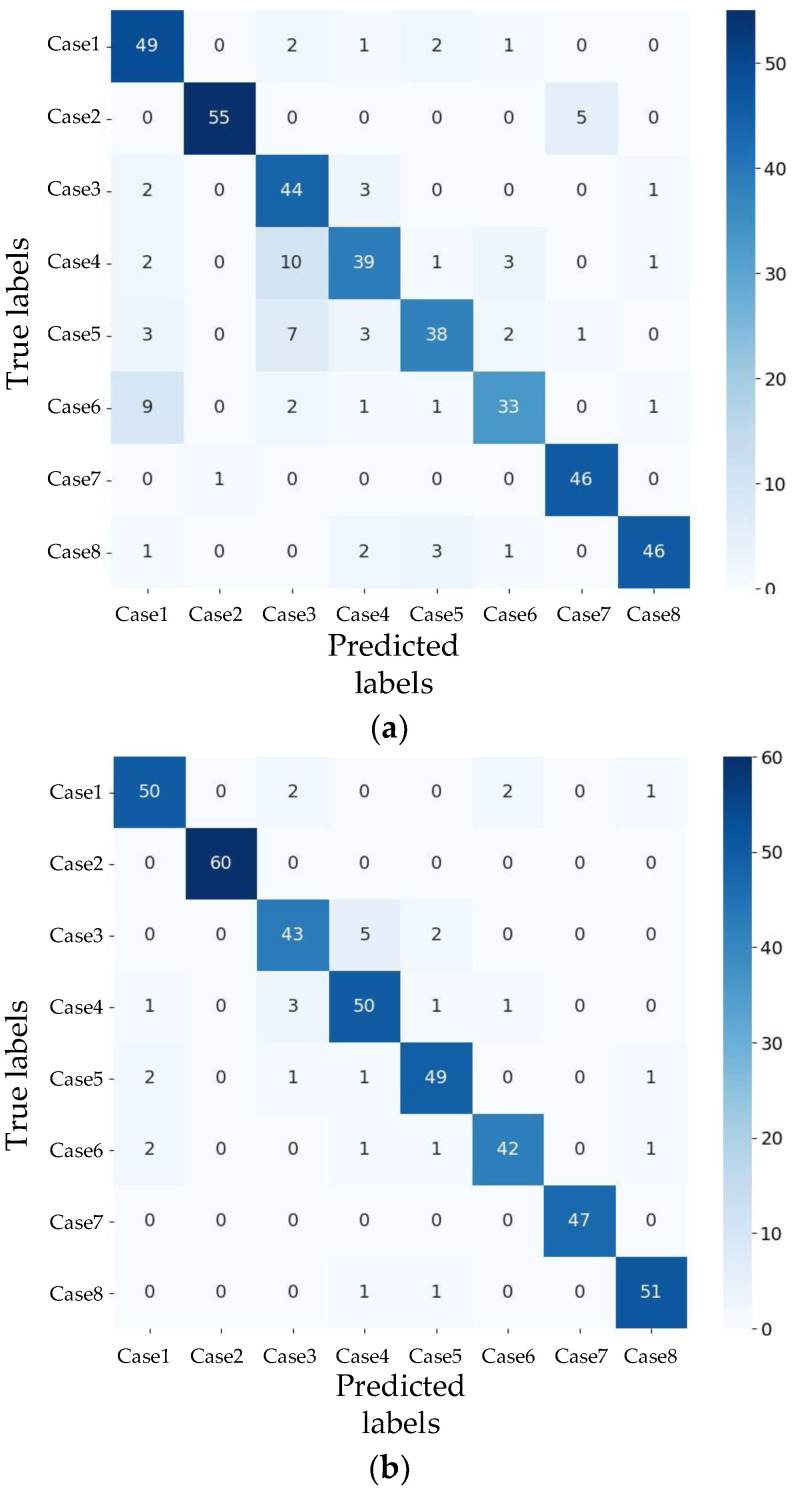
Confusion matrix for different networks: (**a**) AlexNet. (**b**) ResNet.

The confusion matrices for AlexNet and ResNet were plotted to compare recognition performance across different conditions. The confusion matrix for the test accuracy of AlexNet is shown in Figure 11a. It is evident that Case 7 performs well, while Case 4 shows poorer performance. Figure 11b shows the confusion matrix for the test accuracy of ResNet, revealing that Conditions 2 and 7 yield better results, while Case 3 performs less effectively.

### 4.3. Network Robustness Under Noise Conditions

In practical scenarios, external noise inevitably affects collected signals, making it essential to examine how various noise conditions impact the vibration signals collected experimentally. Gaussian white noise was added to the vibration signals collected under each condition. This study evaluates four different noise levels with Signal-to-Noise Ratios (SNRs) ranging from 20 to 2.5 dB to assess the impact of varying SNR levels on the collected vibration signals.

**Figure 12:**
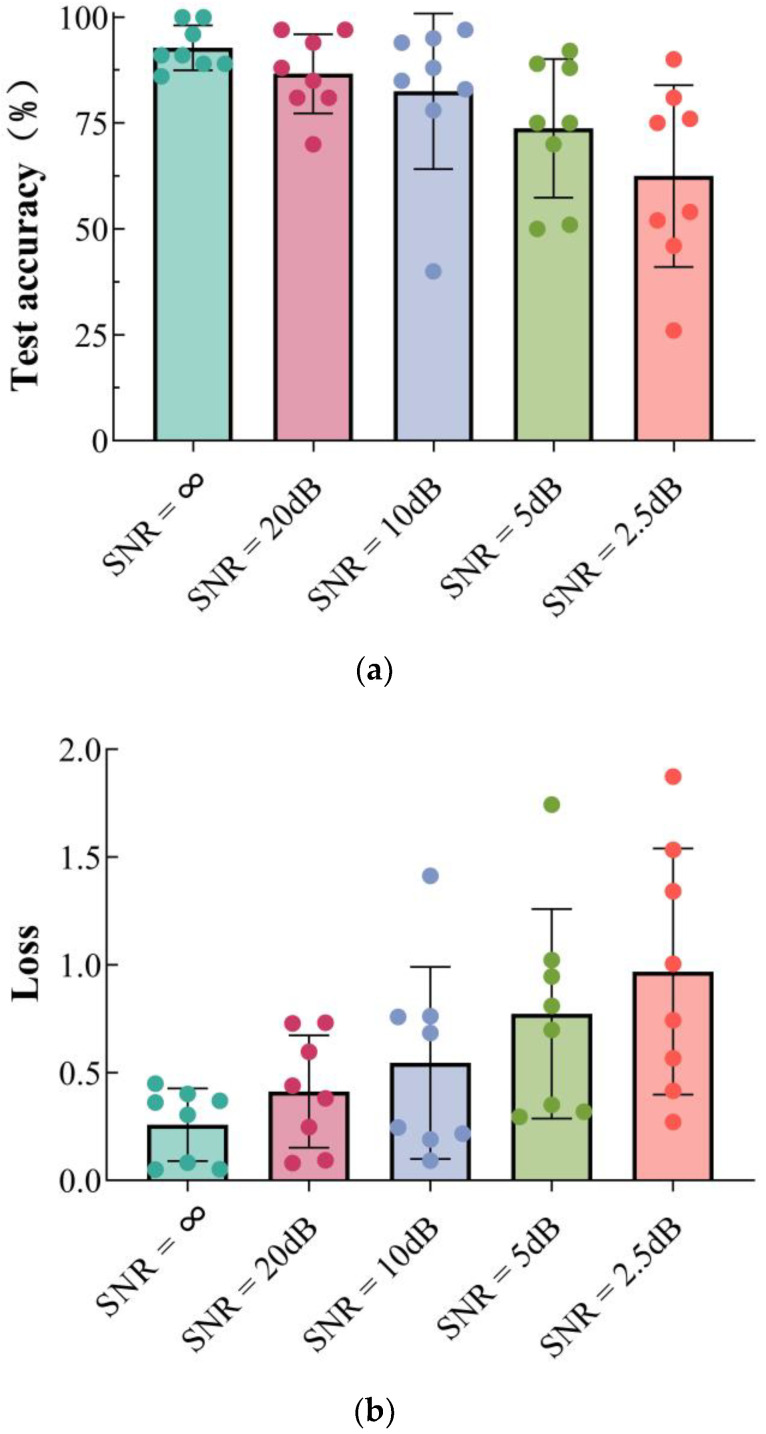
Test accuracy and loss for different Signal-to-Noise Ratio scenarios: (**a**) accuracy; (**b**) loss.

Using the ResNet network as an example, Gaussian white noise was introduced to the experimental signals to compare how varying Signal-to-Noise Ratio (SNR) conditions affect test accuracy and loss. Figure 12a,b shows the test accuracy and loss for the eight damage conditions at different noise levels. Table 3 shows the classification accuracy of specified working conditions.

In Figure 12, circles represent various damage conditions, with radial data indicating the test accuracy and loss of the ResNet network under different noise levels. At first, as noise levels increase, the images become affected by noise across various frequency bands. However, the ResNet model remains capable of learning features from these noisy signals and maintains relatively good damage detection performance. When the Signal-to-Noise Ratio (SNR) is above five dB, the prediction accuracy for each damage condition remains high, and the test loss is relatively low. As the SNR continues to decrease, the test accuracy for most damage conditions declines and test loss rises. This performance decline is due to high-level noise increasingly masking the vibration signals, which hinders the model’s ability to extract initial damage features and results in misidentifications. Despite the reduction in accuracy and the increase in loss with higher noise levels, the ResNet model remains robust and reliable for structural damage identification.

## 5. Conclusions

This study introduces a method for identifying damage in cable-stayed bridges using time–frequency monitoring data. The presented approach improves the accuracy and efficiency of detecting damage in complex bridge structures. A scaled indoor model of a cable-stayed bridge, equipped with a moving cart and track, was developed to gather comprehensive data on beam vibrations under different cable damage scenarios. The accuracy of four neural networks in detecting cable damage was evaluated, and the influence of noise on their performance was analyzed. The main conclusions are as follows:

(1) The acceleration response of the vehicle–bridge interaction system was projected into the time–frequency domain using GADF, preserving the time-dependent and nonlinear characteristics of the time series in the resulting images. This process facilitates the creation of a labeled dataset with 2D time–frequency representations of the signals, enhancing classification and analysis through deep learning methods and improving accuracy.
(2) The ResNet was demonstrated to have the best performance in terms of the damage identification accuracy and convergence speed, achieving a higher accuracy and faster convergence compared to other networks.
(3) As the SNR decreases from 20 dB to 2.5 dB, ResNet’s prediction accuracy declines from 86.63% to 62.5%. Despite this, tests across eight damage conditions under different noise levels demonstrated that the ResNet model maintains strong robustness and reliability in identifying structural damage.

The experimental results demonstrate that the proposed method effectively identifies cable damage in the experimental cable-stayed bridge structure. However, this study focuses on a cable structure without accounting for the complexity of bridge environments. Future research should extend the application of this method to actual bridges.

## Figures and Tables

**Table 1:** Cable forces of the cable-stayed bridge specimen.

Cable Number	S1	S2	S3	S4	S5	S6	S7	S8
Cable force/N	12.3	17	20.7	37.1	29.5	24.8	15.3	13.4

**Table 2:** Classification table of working conditions.

Scenario	Damage Location	Damage Levels
Case 1	Intact	/
Case 2	S1	30%
Case 3	S3	30%
Case 4	S5	30%
Case 5	S1, S4	30%
Case 6	S2, S5	30%
Case 7	N2, N4	30%
Case 8	N5, N6	30%

**Table 3:** Classification accuracy of working conditions.

SNR	0 dB	20 dB	10 dB	5 dB	2.5 dB
Accuracy	92.75%	86.63%	82.50%	73.75%	62.5%
Loss	0.26	0.41	0.55	0.77	0.97

## Data Availability

Data will be made available on request.
